# ﻿A new species of *Neotoxoscelus* Fisher, 1921 (Coleoptera, Buprestidae) from South Korea, with an updated checklist

**DOI:** 10.3897/zookeys.1114.81696

**Published:** 2022-07-20

**Authors:** Donguk Kim, Sang Jae Suh

**Affiliations:** 1 School of Applied Biosciences, College of Agricultural and Life Sciences, Kyungpook National University, Daegu 41566, Republic of Korea Kyungpook National University Daegu Republic of Korea; 2 Institute of Plant Medicine, Kyungpook National University, Daegu 41566, Republic of Korea Kyungpook National University Daegu Republic of Korea

**Keywords:** Buprestidae, jewel beetle, *Neotoxoscelus*, new species, South Korea, taxonomy

## Abstract

*Neotoxosceluspetilus***sp. nov.** is described from South Korea. *Neotoxoscelus* Fisher, 1921 is also a new generic record for South Korea. A key to the *Neotoxoscelus* species and an updated checklist are provided.

## ﻿Introduction

The genus *Neotoxoscelus* Fisher, 1921 (Coleoptera, Buprestidae, Agrilinae, Coraebini, Toxoscelina) is a small group, with only seven known species globally. Most of these species are distributed in Oriental Region: *N.aeneiventris* Fisher, 1930, *N.bakeri* Fisher, 1921, *N.corporaali* Obenberger, 1922, *N.kurosawai* (Hattori, 1990), *N.luzonicus* Fisher, 1921, and *N.ornatus* Fisher, 1930, with only *N.kerzhneri* (Alexeev, 1975) reported from the Palaearctic Region (Bellamy 2008; Kubáň 2016). Although there is a record of *N.kurosawai* collected from the leaves of a *Quercus* tree standing at the edge of a forest at the foot of a hill (Hattori 1990), the detailed biology of members of this genus is unknown.

Until now, only one species of the subtribe Toxoscelina, *Toxoscelusauriceps* (Saunders, 1873), had been reported from South Korea (Kurosawa et al. 1985; ESK and KSAE 1994; Löbl and Smetana 2006; Paek et al. 2010; Hong and Lee 2014; Kubáň 2016; NIBR 2019). Recently, we conducted a study of an unknown species of Toxoscelina and confirmed it as a new species belonging to the genus *Neotoxoscelus*. As a result, a total of two species within two genera of Toxoscelina are known for the fauna of South Korea. In this paper, *Neotoxosceluspetilus* sp. nov. is described as new to science, and the genus *Neotoxoscelus* is reported for the first time from South Korea.

## ﻿Materials and methods

**Figure 1.**
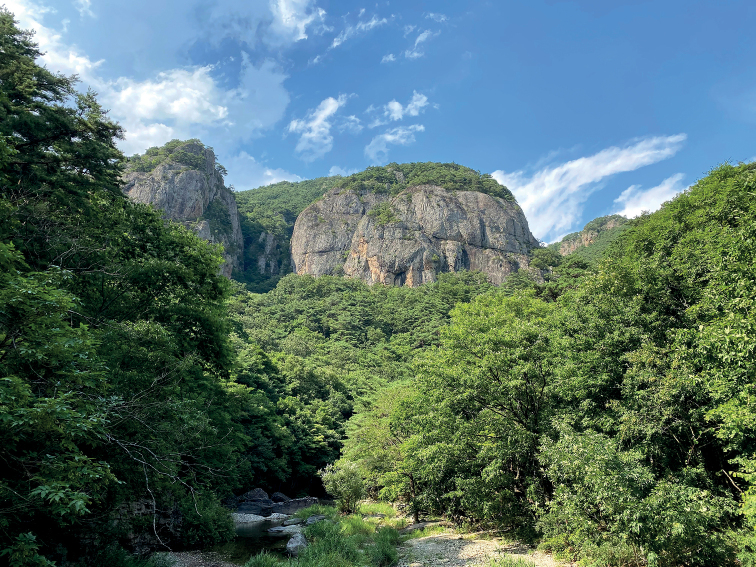
Habitat of *Neotoxosceluspetilus* sp. nov.; the type locality in Cheongsong-gun, Gyeongsangbuk-do province.

The specimen was collected in Mt. Juwangsan, Cheongsong-gun, Gyeongsangbuk-do province, South Korea (Fig. 1) by sweeping, and is preserved in dried conditions. A taxonomic study was conducted using either a stereoscopic microscope (Olympus SZX16) and a compound microscope (Olympus BX50) for accurate identification. An Olympus camera (DP 71) was used to photograph the specimen, and Adobe Photoshop 21.2.0 (Adobe Systems Inc.) was used to edit the captured images. For dissection of the male genitalia, the specimen was softened in 80 °C distilled water using 10% potassium hydroxide for 10 minutes. Then, the last abdominal tergite (pygidium) and the abdominal sternites were opened using dissecting tweezers. The softened specimen was mounted on a card point and the male genitalia were placed in a microtube with glycerin for long-term preservation. The type specimen is deposited in the insect collection of the Systematic Entomology Laboratory, College of Agriculture and Life Sciences, Kyungpook National University (**KNU**), Daegu, Gyeongsangbuk-do, South Korea.

### ﻿Measurement criteria

Body length: distance from apex of head to apex of elytra.

Elytral length: distance from anterior margin to apex of elytra.

Elytral width: width of both elytra at widest point.

Pronotal length: length of pronotum along midline.

Pronotal width: width of pronotum at widest point.

## ﻿Taxonomy

**Family Buprestidae Leach, 1815**

**Subfamily Agrilinae Laporte, 1835**

**Tribe Coraebini Bedel, 1921**

### ﻿Subtribe Toxoscelina Majer, 2000

#### ﻿Genus Neotoxoscelus

Taxon classificationAnimaliaColeopteraBuprestidae

Fisher, 1921

62FECCD4-417C-5F27-B4DE-E950021C3000

Neotoxoscelus Fisher, 1921: 418. Type species: Neotoxoscelusbakeri Fisher, 1921 (fixed by original designation).

##### Diagnosis.

Pronotum convex; inner margin of all tibiae almost straight; no space between tibiae and femora when closed; apical margin of pygidium with a short spinous process in middle part (Fisher 1921).

#### ﻿ Neotoxoscelus petilus sp. nov.

Taxon classificationAnimaliaColeopteraBuprestidae

9160B56F-68B0-538C-8DBC-599E26B69682

https://zoobank.org/8141964D-10A7-4672-8C08-C542815299BB

**Figure 2.**
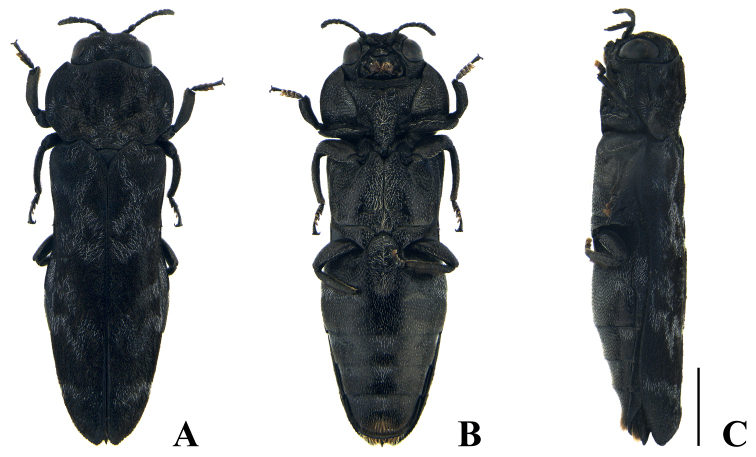
Habitus of *Neotoxosceluspetilus* sp. nov., holotype, male **A** dorsal view **B** ventral view **C** lateral view. Scale bar: 1.0 mm.

**Figure 3.**
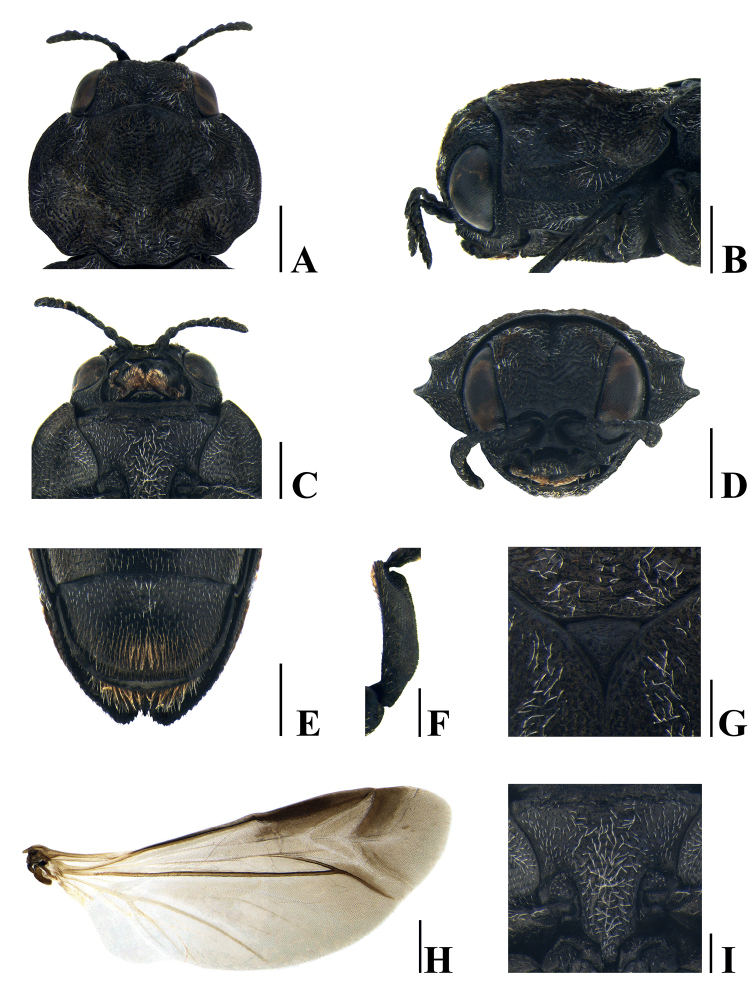
*Neotoxosceluspetilus* sp. nov., holotype, male **A** pronotum, dorsal view **B** ditto, lateral view **C** prosternum **D** head, frontal view **E** sternite V and spinous process, ventral view **F** protibia, dorsal view **G** scutellum **H** hind wing **I** prosternal process. Scale bars: 0.5 mm (**A, H**); 0.4 mm (**B–E**); 0.2 mm (**F–G, I**).

**Figure 4.**
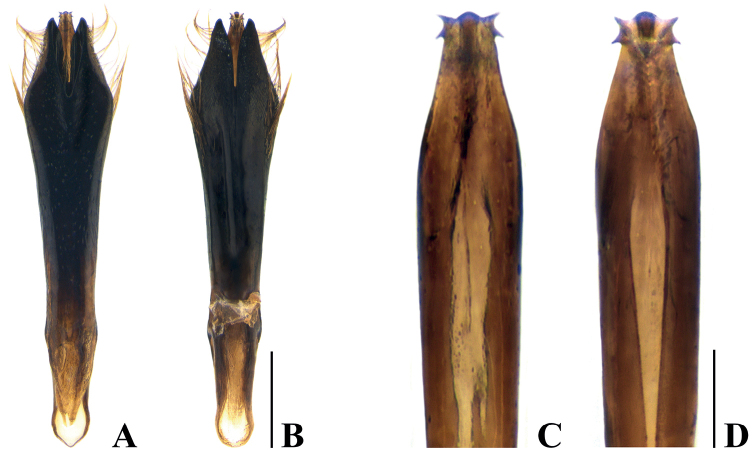
Male genitalia structures of *Neotoxosceluspetilus* sp. nov. **A** genitalia, dorsal view **B** ditto, ventral view **C** apical part of median lobe, dorsal view **D** ditto, ventral view. Scale bars: 0.4 mm (**A–B**); 0.1 mm (**C–D**).

Figs 2 , 3 , 4 Korean name: 가시밤나무비단벌레

##### Differential diagnosis.

This new species is most similar to *N.kerzhneri* (Alexeev, 1975) among its congeners. It can be distinguished from *N.kerzhneri* based on the following combination of morphological characters: body slender; lateral margin of pronotum slightly curved and narrowed with somewhat distinct sinuation, basal angle rounded; scutellum wider than long; elytra widest at humerus, longer than wide: W/L = 2.60 (width at humerus), W/L = 2.40 (width including laterosternites); median lobe of male genitalia with two pointed apical denticles on each side and lateral margin slightly narrowed. In contrast, *N.kerzhneri* has the following aspects: stout body; lateral margin of pronotum more roundly curved and rectilinearly narrowed, without sinuation, basal angle angulated; scutellum longer than wide; elytra widest at posterior 5/9, longer than wide: W/L = 2.35–2.40 (width at humerus), W/L = 2.04–2.16 (width including laterosternites); median lobe of male genitalia without apical denticles on each side and lateral margin drastically narrowed (Alexeev 1975; Emeljanov and Medvedev 2008).

##### Description.

**Male** (Fig. 2): body length: 5.3 mm, width: 1.7 mm.

***Body*** slender and elongate, widest at pronotum, dorsal side black without gloss, ventral side black and somewhat glossy. ***Head*** (Fig. 3A–D) small and convex, covered with short, recumbent, whitish and dark brownish hairs arranged moderately on vertex and sparsely on frons; longitudinal sulcus rather distinctly dented at vertex and shallowed towards frons; eye large and longitudinally elongate; clypeus somewhat oval and narrow; antenna short; 1^st^ antennomere stout, cylindrical; 2^nd^ antennomere stout, fusiform, and slightly shorter than 1^st^; 3^rd^ and 4^th^ antennomeres almost cylindrical, remarkably narrower than 2^nd^ and nearly equal in length; 5^th^–10^th^ antennomeres serrated and dilated, each nearly as long as wide; 11^th^ antennomere narrowed to apex. ***Pronotum*** (Fig. 3A, B, D) rugose and punctate, covered with recumbent, dark brownish and whitish hairs, widest at midpoint, wider than long: L/W = 1.45 (width at midpoint), L/W = 1.30 (width at basal fourth); anterior margin arcuately bisinuate and convex at mid part; lateral margin slightly curved from apex to anterior 3/4 and narrowed, with somewhat distinct sinuation; posterior margin arcuately bisinuate and weakly sinuated at mid part; basal angle rounded; lateral carina strongly elevated, arcuately extending from near anterior margin to just posterior to midpoint and exteriorly converging to lateral margin; carinal interspace dented; disc uneven, with laterobasal depressions and projected from base to apex. ***Scutellum*** (Fig. 3G) rugose, wider than long (L/W = 1.20), nearly an equilateral triangle; anterior margin slightly convex; lateral margin weakly concave. ***Elytra*** (Fig. 2A, C) irregularly rugose and punctate, covered with recumbent, dark brownish, inconspicuous hairs, longer than wide (W/L = 2.60, including laterosternites 2.40), slightly narrower than maximum pronotal width and widest at humerus; anterior margin strongly and narrowly bisinuate; humeral angle rounded; lateral margin slightly concave from humerus to posterior 2/5 and narrowed almost rectilinearly, tapered and slightly arcuate to apex, with feeble marginal teeth; disk slightly flattened; ornamentation remarkable, with whitish hairs arranged as follows: 1^st^ band somewhat narrow, strongly curved interiorly; 2^nd^ band eye-shaped, longitudinally subrectangular, extended laterally, linked with 1^st^ band apically; 3^rd^ band in posterior 3/5 transversely short and zigzagged; 4^th^ band in posterior 2/5 transversely long and zigzagged, forming a triangular ring with 3^rd^ band; 5^th^ band feebly zigzagged, somewhat far from 4^th^. ***Laterosternites*** (Fig. 2A, C) ornamented with recumbent, whitish hairs in posterior 3/5 of elytra, seemingly linked with zigzagged band of elytra. ***Hindwing*** (Fig. 3H) sclerotized with markedly reduced venation. ***Prosternum*** (Fig. 3C) slightly convex, rugose and punctate, covered with recumbent, short, whitish hairs, longer and thicker toward middle. ***Prosternal process*** (Fig. 3I) feebly convex, composed of subrectangular microsculpture, rather concentrically rugose, covered with long and thick hairs; lateral margin slightly concave, arcuately narrowed behind anterior coxal cavities, angulated subapically, rounded at apex. ***Abdominal sternites*** (Figs 2B, 3E) black and somewhat glossy, covered with overall sparsely recumbent, short, yellowish and whitish hairs; sternite I longest, following sternites each gradually shorter than preceding sternite, except sternite V; lateral part of sternite I and anterolateral part of sternite II remarkably congregated, with rather long, white hairs; anteromedian and anterolateral parts of sternites III–V with remarkable whitish hairs and slightly attenuated toward the apex; sternite V with long and brownish hairs posteriorly, densely congregated in middle. ***Legs*** somewhat short; protibia (Fig. 3F) alveolate, widest at posterior 1/3, outer margin slightly convex and crenulated, inner margin slightly curved, with dense yellowish hairs at anterior part and somewhat sparse hairs at posterior part, oblique grooves elongated rather distinctly dented at apex and shallowed posteriorly; all tarsi with brownish tarsal lamella, each tarsomere gradually widened anteriorly. ***Genitalia*** (Fig. 4) longer than wide (W/L = 4.75), widest at anterior 1/5, sparsely punctuated dorsally, apical part with oblique grooves ventrally; paramere obliquely narrowed from widest part to apex, with long, yellowish hairs; median lobe with two pointed apical denticles on each side and lateral margin slightly narrowed to apex.

**Female.** Unknown.

##### Type material.

***Holotype*.** ♂, South Korea, Gyeongsangbuk-do province, Cheongsong-gun, Budong-myeon, Sangui-ri, Mt. Juwangsan, ca 550 m alt., 36°23'21.7"N, 129°10'06.9"E, sweeping, 26. VII. 1984, S.J. Suh Coll. (KNU).

##### Host plants.

Unknown.

##### Distribution.

South Korea, Gyeongsangbuk-do province.

##### Etymology.

The specific epithet refers to the slender body shape of the new species.

### ﻿Key to the *Neotoxoscelus* species

**Table:** 

1	Dorsal side of body shining dark brown with strong violaceous tinge	***N.bakeri* Fisher, 1921**
–	Dorsal side of body black	**2**
2	Elytra with a long acuate apical spine	***N.aeneiventris* Fisher, 1930**
–	Elytra without a long acuate apical spine	**3**
3	Disk of elytra slightly convex; apex of elytra obliquely truncated	***N.ornatus* Fisher, 1930**
–	Disk of elytra slightly flattened; apex of elytra not truncated	**4**
4	Apex of elytra with remarkably strong marginal teeth	***N.corporaali* Obenberger, 1922**
–	Apex of elytra with unremarkable marginal teeth	**5**
5	Dorsal side of body with feeble bluish tinge; base of elytra distinctly narrower than widest part of pronotum; elytral ornamentation in posterior 1/2 with a transversely zigzagged band	***N.luzonicus* Fisher, 1921**
–	Dorsal side of body without feeble bluish tinge; base of elytra slightly narrower than widest part of pronotum; elytral ornamentation in posterior 1/2 with two transversely zigzagged bands	**6**
6	Ventral side of body with faint violaceous tinge; paramere of male genitalia convexly narrowed to apex; median lobe concave apically	***N.kurosawai* (Hattori, 1990)**
–	Ventral side of body without violaceous tinge; paramere of male genitalia slightly concavely narrowed to apex; median lobe convex apically	**7**
7	Lateral margin of pronotum more roundly curved and rectilinearly narrowed, without sinuation, basal angle angulated; elytra widest at posterior 5/9; median lobe of male genitalia without apical denticles on each side, lateral margin drastically narrowed	***N.kerzhneri* (Alexeev, 1975)**
–	Lateral margin of pronotum slightly curved and narrowed, with somewhat distinct sinuation, basal angle rounded; elytra widest at humerus; median lobe of male genitalia with two pointed apical denticles on each side, lateral margin slightly narrowed	***N.petilus* sp. nov.**

### ﻿Updated checklist of *Neotoxoscelus* species

***Neotoxoscelusaeneiventris* Fisher, 1930**

**Distribution.** Malaysia (Pahang).

***Neotoxoscelusbakeri* Fisher, 1921**

**Distribution.** Philippines (Mindanao).

***Neotoxosceluscorporaali* Obenberger, 1922**

**Distribution.** Indonesia (Sumatura).

***Neotoxosceluskerzhneri* (Alexeev, 1975)**

**Distribution.** China (Liaoning, Shanghai, Shanxi), Mongolia (Dornogovi).

***Neotoxosceluskurosawai* (Hattori, 1990)**

**Distribution.** Taiwan.

***Neotoxoscelusluzonicus* Fisher, 1921**

**Distribution.** Philippines (Luzon).

***Neotoxoscelusornatus* Fisher, 1930**

**Distribution.** Malaysia (Sabah).

***Neotoxosceluspetilus* sp. nov.**

**Distribution.** South Korea.

## Supplementary Material

XML Treatment for Neotoxoscelus

XML Treatment for Neotoxoscelus petilus
